# Impact of meningitis on intelligence and development: A systematic review and meta-analysis

**DOI:** 10.1371/journal.pone.0175024

**Published:** 2017-08-24

**Authors:** Deborah Christie, Harunor Rashid, Haitham El-Bashir, Faye Sweeney, Tim Shore, Robert Booy, Russell M. Viner

**Affiliations:** 1 UCL Institute of Epidemiology & Health Care, London, United Kingdom; 2 National Centre for Immunisation Research and Surveillance of Vaccine Preventable Diseases (NCIRS), The Children’s Hospital at Westmead, Westmead, Australia; 3 Discipline of Paediatrics and Child Health, Sydney Medical School, The University of Sydney, Sydney, NSW, Australia; 4 Rehabilitation Department, Al Jalila Children Specialty Hospital, Dubai, UAE; 5 UCL Institute of Child Health, London, United Kingdom; University Children's Hospital Tuebingen, GERMANY

## Abstract

**Background:**

We undertook a systematic review and meta-analysis to address the question “what is the impact of meningitis on IQ and development.”

**Methods:**

Search: conducted using standardized search terms across Medline, PsychInfo and EMBASE to 06/2014. Eligibility: human studies of any infectious aetiology of meningitis reporting IQ or infant developmental age or stage outcomes. Quality: Centre for Evidence Based Medicine, Oxford, quality tools. Analysis: random effects meta-analysis by organism.

**Results:**

39 studies were included in the review, 34 providing data on IQ (2015 subjects) and 12 on developmental delay (382 subjects). Across all bacterial organisms, meningitis survivors had a mean IQ 5.50 (95% CI: -7.19, -3.80; I^2^ = 47%, p = 0.02) points lower than controls. IQ was significantly lower than controls for Neisseria meningitides (NM: 5 points) and *Haemophilus influenzae* b (Hib: 6 points) but not in viral meningitis, with only single studies included for *Streptococcus pneumoniae* (SP) and group B streptococcus (GBS). The pooled relative risk (RR) for low IQ (IQ<70) in survivors of bacterial meningitis compared with controls was 4.99 (95% CI: 3.17, 7.86) with no significant heterogeneity (I2 = 49%, p = 0.07). Developmental delay of approximately 0.5SD was reported in studies of bacterial meningitis but no delay in the only study of viral meningitis.

**Conclusions:**

We found moderate evidence that surviving bacterial meningitis has a deleterious impact on IQ and development but no evidence that viral meningitis had meaningful cognitive impacts. Survivors of bacterial meningitis should be routinely offered screening for cognitive deficits and developmental delay in addition to hearing loss.

## Background

The epidemiology of bacterial meningitis has changed substantially over the past century. The use of conjugate vaccines over the last couple of decades has drastically changed the epidemiology of the disease in high income countries, leading to ‘near elimination’ of *Haemophilus influenzae* b [Hib] meningitis as well as serogroup type C meningococcal meningitis.[1] However, worldwide bacterial meningitis remains a major cause of morbidity and mortality, causing annually over 303,000 deaths and 2,628,000 years lived with disability.[2, 3] In Asia and Africa about one fifth to a quarter of survivors suffer from long-term sequelae.[4]

In the era of conjugate vaccines, viral rather than bacterial meningitis is the most common form of meningitis in high income countries. Amongst bacterial causes, the previously less common group B streptococcus (GBS) is now the most common pathogen causing purulent meningitis.[5] Improvements in management and antibiotics now limit mortality and serious sequelae in those bacterial cases that do occur,[6] whilst sequelae of aseptic meningitis are usually limited to subtle neurocognitive problems.[7]

Systematic reviews of the outcomes of meningitis are limited to bacterial meningitis and have not assessed in detail on the impact of meningitis on key neurocognitive outcomes such as intelligence and development. Intelligence Quotient (IQ) is a construct used within standardized tests as a measure of an individual's intelligence level. Current IQ tests measure two primary components; verbal IQ (VIQ) relates to verbal and language abilities, reasoning and arithmetic skills and verbal memory. Performance IQ (PIQ) in contrast relates to visuospatial and performance skills. In young children or in those who cannot complete formal IQ tests, concepts of developmental delay (DD) are frequently used. DD is defined as delay in meeting developmental milestones in one or more domains of development. Key domains are similar to those used in measuring IQ, and include cognition, language, visual problem solving, motor development and social-emotional development. There is not surprisingly a major overlap between the two constructs.

A systematic review of sequelae due to bacterial meningitis in African children did not report on IQ.[8] The most recent and comprehensive systematic review of disabling sequelae from bacterial meningitis reported only on the prevalence of major cognitive deficits, defined as IQ <70.[4]

Overall intelligence, measured by IQ, is one of the strongest predictors of an individual’s future life chances, material and subjective well-being and health.[9, 10] Even small deficits in IQ following meningitis can impair life-chances and educational attainments.[11] IQ can be difficult to assess in very young children, a majority of those studied in meningitis outcome studies, and studies may instead estimate DD.

We undertook a systematic review and meta-analysisof the neurocognitive outcomes of meningitis of all causes in humans published in the last 50 years to address the question “what is the impact of meningitis on IQ and development”.

## Methods

Data for this review were obtained from a wider review of the complications and sequelae of meningitis.

### Search

The search terms used aimed to be as inclusive as possible to identified any complications or sequelae of meningitis. We undertook a computerised search of the MEDLINE (6/1964–06/2014) database using the MeSH terms: [“Meningitis” AND (“Meningitis/complications” OR “Meningitis/psychology” OR “Meningitis/rehabilitation”)]. We also searched PsychoINFO (1955-06/2014) and EMBASE (1982-06/2014) using similar terms. Eligible studies were observational studies of long term outcomes subsequent to meningitis meeting the following inclusion and exclusion criteria. The reference lists of identified articles were then hand searched for further relevant studies. We also hand-searched identified systematic reviews.[4, 8, 12]

### Eligibility

For the wider review, eligibility criteria were human studies of any age-group; laboratory proven meningitis of any infectious aetiology together with meningococcal septicaemia; studies must address outcomes/sequelae or complications of meningitis other than death or acute complications; time-scale: Outcomes or follow-up reported ≥1 month after meningitis; study type: prospective and retrospective cohort, case–control, or cross-sectional studies of outcomes following meningitis; and language of publication was English or a published English translation was available. We excluded studies involving only acute complications (<1 month after onset); single or multiple case reports; papers published before 1955; papers published in languages other than English (except if published English translation) and meningitis in immunosuppressed populations.

### Study selection

Articles identified by the electronic searches were examined by 1 of 2 reviewers. Articles that clearly did not meet eligibility criteria were rejected on initial review. Articles marked for potential inclusion were than obtained electronically or in paper copy, and assessed again for inclusion. Those included studies deemed to potentially meet inclusion criteria were appraised. A form was used to record all details of the papers reviewed. Where repeated assessments of a cohort had been reported at different times, only the latest and most comprehensive assessment was included in the review.

### Eligibility for this review

For this paper we applied further eligibility criteria. Eligible studies were those that assessed IQ or infant developmental age or stage using validated instruments and reported any of the following outcomes:

a. Intelligence: mean full-scale, performance IQ (PIQ) or verbal IQ (VIQ) reported as standardized scores (mean 100, SD 15) or proportions with low IQ (<70 i.e. >2 standard deviations (SDs) below mean)

b. Infant development: outcomes of interest were developmental performance in motor, language or cognitive domains compared with normative data, providing a measure of DD.

We did not include studies which reported IQ or developmental outcomes on subsets of meningitis survivors e.g. those with or without neurological sequelae. We did not include studies which reported only certain IQ subscales. For case-control studies, we only included studies which reported details of control recruitment and matching.

### Quality

Each publication was reviewed and graded by using Evidence Based Medicine tools devised by the Centre for Evidence Based Medicine, Oxford (http://www.cebm.net). Studies were classified as retrospective or prospective cohort studies (depending on whether meningitis preceded or anteceded cohort recruitment) or case-control studies (if a retrospectively defined sample of meningitis subjects were compared with a healthy control group).

### Data extraction

Data were extracted by one reviewer and then checked by a clinical neuropsychologist (DC) to ensure accuracy.

### Analysis

Descriptive analyses were first undertaken of the distribution of each outcome by causative organism. Where data allowed, random effects meta-analyses were undertaken in Stata13 (StataCorp; College Station, TX) using the *metan* commands. The level of significance used was the 0.05 level. Study weights were assigned automatically by Stata. Where IQ SDs were not reported, these were obtained from standard errors, confidence intervals, t values or p values that relate to the differences between means in two groups. Where SD values for IQ were not available through the above methods, an SD of 15 was substituted as IQ scales are normed to have a mean of 100 with an SD of 15. Where there were serial published follow-up studies on the same cohort, we only included the latest assessment study. Where papers presented case-control findings both unmatched and matched, the matched analyses were used in this review.

We first conducted meta-analyses across all bacterial causes. Separate metanalyses were conducted by organism only where ≥3 studies were available in each category. The DerSimonian and Laird Q test was performed to assess the degree of heterogeneity between studies, and the I^2^ statistic was used to describe the percentage of total variation across studies due to heterogeneity.

### Reporting

The QUORUM (Quality of Reporting of Meta-analyses) guideline was used for reporting our review.[13]

## Results

**Fig 1:**
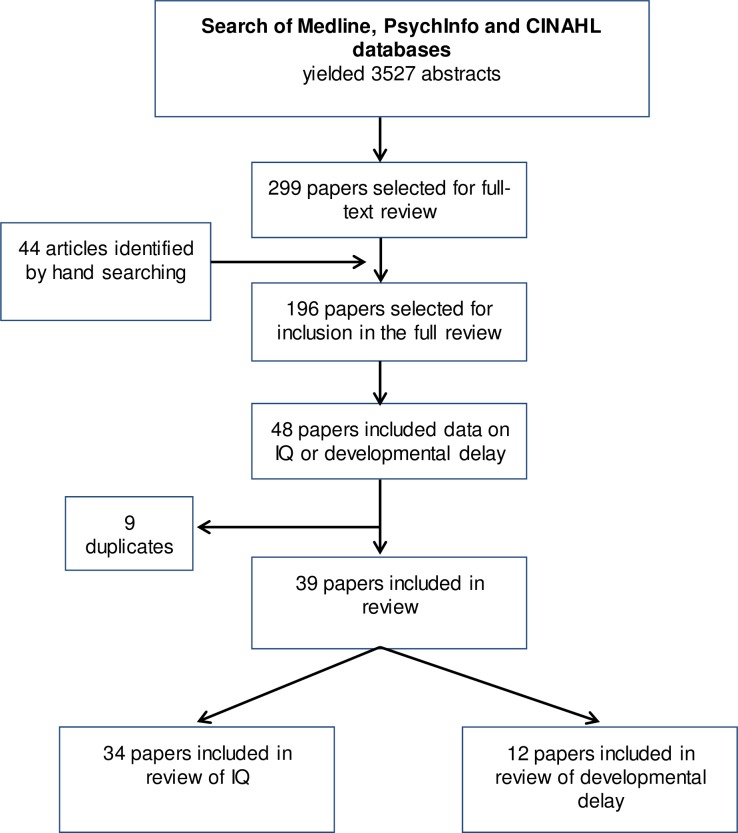
Study selection flowchart for review.

Searches yielded 3527 articles, of which 299 papers were selected for full-text reviewing and 196 were included in the wider review of all meningitis complications and sequelae. Of these, 48 papers included estimates of IQ and / or developmental delay meeting our additional eligibility criteria, of which 9 were duplicate publications on the same cohorts, thus 39 papers were included in these analyses. 34 studies provided data on IQ including 2015 survivors, and 12 studies on the prevalence of DD were included representing 382 survivors (see Fig 1 for search flow chart). Characteristics of the included papers are shown in S1 Table. Only 7 studies came from low- or middle-income countries.

The vast majority of studies were of those with meningitis in early childhood, who were then followed up later in childhood and adolescence. There was only one study each of adolescent onset meningitis (Borg et al. 2009) and of meningitis in adults (Merkelbach et al. 2000).

### IQ data

Of the 34 IQ studies, 9 studies (26%) were retrospective cohort studies, 1 (3%) was a prospective cohort study and 24 (71%) were case-control studies. 8 (24%) studies recruited cases from population-based samples, with the remaining 26 (76%) being clinical series recruited from one or more hospitals. Of the case-control studies, most matched controls for age and sex, with the majority matching controls on the basis of socioeconomic status, either by using sibling controls or by matching population controls.

In terms of organism, 8 (24%) studies included any bacterial cause with an additional 3 (9%) studies of neonatal bacterial meningitis. 8 (24%) studies were of *Haemophilus influenzae* b (Hib), 3 (9%) of *Streptococcus pneumoniae* (SP) and 1 (3%) of Hib and SP, 4 (12%) studies were of *Neisseria meningitides* (NM), 2 (6%) of group B streptococcus (GBS), 2 (6%) of tuberculosis (TB) and 3 (9%) studies were of viral meningitis.

Mean full-scale IQ in meningitis survivors was reported in 23 (68%) studies, with 19 (56%) reporting the prevalence of low IQ defined as IQ <70 and 9 (26%) reporting mean VIQ or PIQ.

Study quality: 2 studies were graded as providing level 2a evidence, 20 studies as level 3b and 12 studies as level 4.

#### Mean IQ

Table 1 shows the characteristics of studies reporting mean IQ and proportions with low IQ by organism. The overall mean full-scale IQ in the whole group across all organisms was 97.1 (n = 26; SD 7.2; range 79 to 110). The mean and median of all organism groups were within 1 standard deviation of the normal IQ range, although no studies reported mean IQ in TB meningitis.

**Table 1:** Full scale IQ: Mean IQ and proportions with low IQ (≤70) by organism.

	Full-scale IQ			IQ <70		
	N of studies	Mean (SD)	Range	N of studies	Mean % (SD)	Range
Bacterial all-cause	6	97.2 (7.3)	(88.8, 110)	4	9.3 (3.4)	(6, 13.5)
Neonatal bacterial all-cause	1	90 (-)	-	3	27.3 (10.9)	(16.7, 38.5)
*Neisseria meningitides* (NM)	4	97.2 (4.5)	(92.9, 102.1)	1	0.8 (-)	-
*Haemophilus influenzae* b (Hib)	7	94.6 (9.9)	(79, 108)	5	11.5 (10.5)	(1, 24.4)
*Streptococcus pneumoniae (SP)*	1	102.4 (-)	-	2	25.6 (22)	(10, 41.2)
*Group B streptococcus* (GBS)	1	101.9 (-)		1	15.0 (-)	-
TB	0	-	-	2	26.3 (24.2)	(9.2, 43.4)
Viral	3	101.6 (3.2)	(97.9, 104)	1	3.03 (-)	-

Notes

For single studies, the mean indicates the published value. SD and range only calculated where there are >1 study.

No studies of all cause meningitis provided quantitative data in either category.

For single studies, the mean indicates the published value. SD and range only calculated where there are >1 study

**Fig 2:**
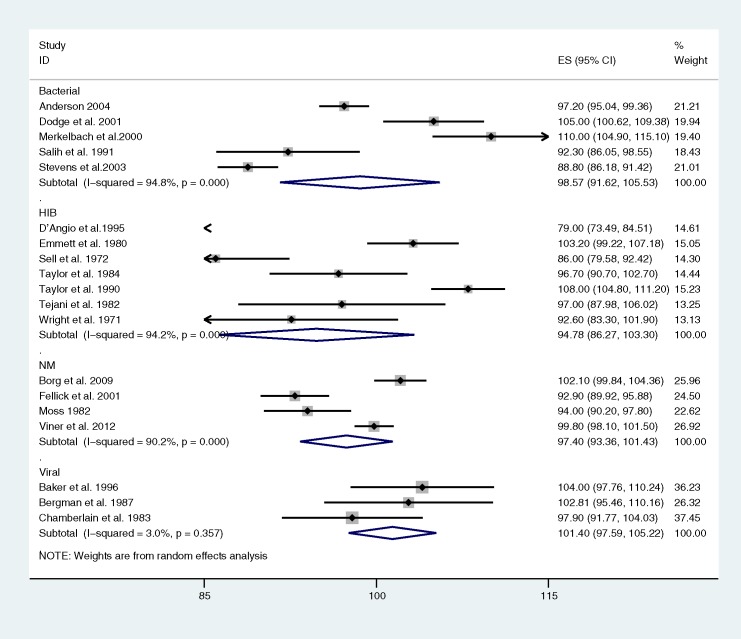
Forest plot of meta-analysis of mean IQ estimates by organism. Abbreviations: *Haemophilus influenzae* b (Hib), *Streptococcus pneumoniae* (SP) *Neisseria meningitides* (NM), group B streptococcus (GBS)

Meta-analysis of mean IQ in survivors across studies was undertaken where more than 3 studies per category were available (Fig 2). Mean IQ estimates appeared highest for viral meningitis and lowest for Hib, but were not significantly different to 100 in any organism. There were insufficient data to repeat meta-analyses including only population-based studies for any organism. There was substantial heterogeneity (I^2^>90%) in all meta-analyses except that for viral meningitis.

**Fig 3:**
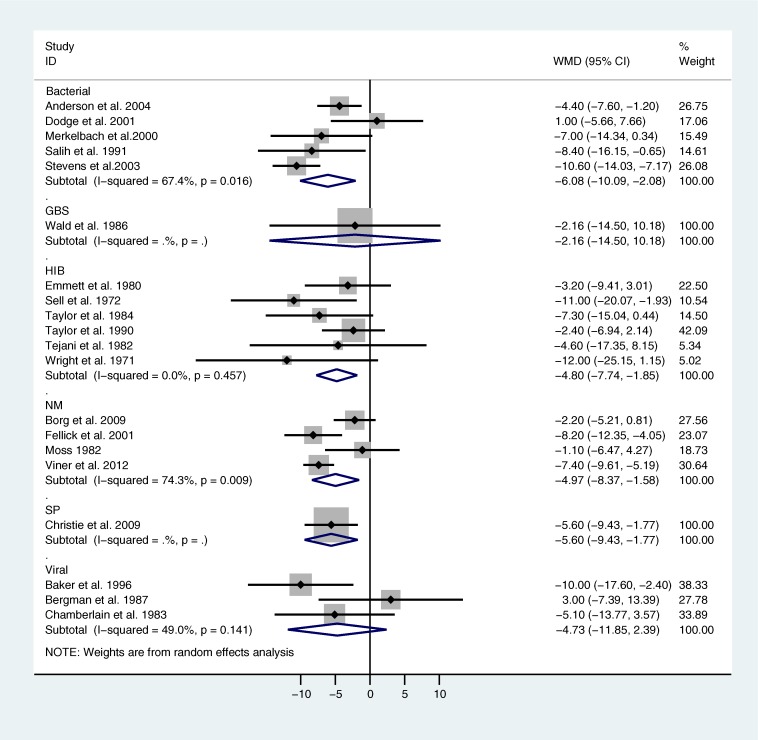
Forest plot of meta-analysis of weighted mean difference in IQ between meningitis survivors and controls by organism. Abbreviations: *Haemophilus influenzae* b (Hib), *Streptococcus pneumoniae* (SP) *Neisseria meningitides* (NM), group B streptococcus (GBS)

Comparison of IQ in survivors compared with controls was reported by 21 studies. Fig 3 shows a Forest plot of a random effects meta-analysis of differences in IQ between survivors and controls. Note that one Hib study (D’Angio et al. 1995) was excluded as this was a study of Navajo Native Indian children in whom there are recognized issues with poor performance on IQ tests related to cultural issues.[14] The single studies found for GBS and SP meningitis are also shown in Fig 3 to demonstrate the data. Survivors of bacterial all-cause, NM and Hib meningitis had a significantly lower IQ than controls, of the order of 5 IQ points for NM and for Hib and 6 for bacterial all-cause meningitis. There was substantial and significant heterogeneity in the bacterial all-cause, and NM but not the Hib analyses. In the single SP study, Christie et al. 2009 reported a significant deficit of 5 IQ points in survivors compared with matched controls, whilst the only GBS study (Wald et al. 1986) was underpowered to detect a difference. A separate meta-analysis across all studies of bacterial organisms (not shown) found that meningitis survivors had a mean IQ 5.50 (95% CI: -7.19, -3.80; I^2^ = 47%, p = 0.02) points lower than controls.

The pooled estimate for viral meningitis was not significantly different to 0, with non-significant heterogeneity.

Only 2 studies, Christie et al (2009) and Viner et al. (2012), reported IQ estimates in meningitis survivors and healthy controls without moderate/severe hearing loss. Both studies reported that exclusion of those with hearing loss did not affect mean IQ estimates or differences with matched controls.

#### Low IQ

Data on proportions of meningitis survivors with low IQ (<70 points) are shown in Table 1. Mean prevalence of low IQ (<70) across all organisms was 17.2% (SD 14.6), range 0.8 to 44.4%. The highest prevalence of low IQ was seen in neonatal, SP and TB meningitis; however exclusion of 1 study (Doctor et al, 2001) which included only low-birth weight neonates resulted in an estimate of 14.4% for neonatal meningitis. It was not possible to perform a meta-analysis of the prevalence of low IQ across studies due to insufficient data.

**Fig 4:**
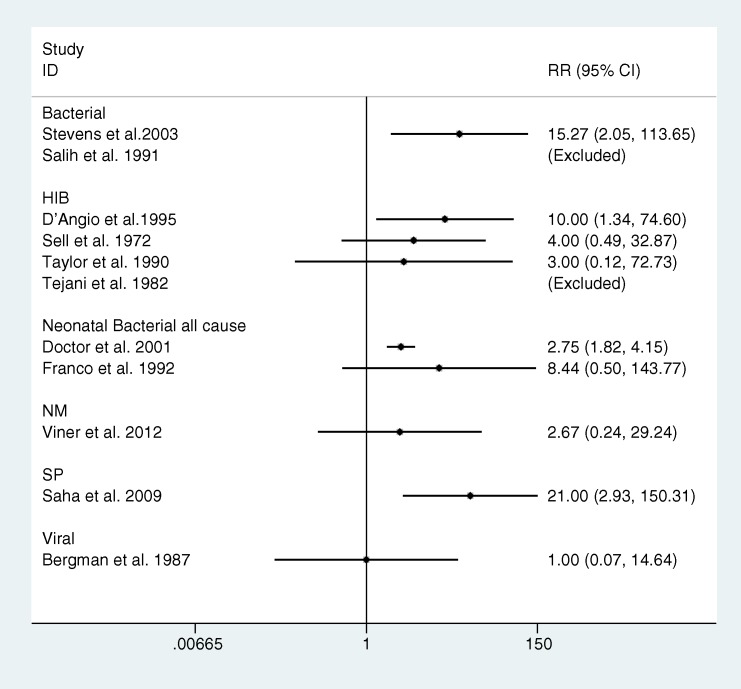
Forest plot of relative risk of low IQ (IQ<70) in meningitis survivors compared with controls by organism. Abbreviations: *Haemophilus influenzae* b (Hib), *Streptococcus pneumoniae* (SP) *Neisseria meningitides* (NM), group B streptococcus (GBS)

Case-control data for low IQ were reported by 11 studies: 2 bacterial all-cause studies, 4 Hib, 1 NM, 2 neonatal, 1 SP and 1 viral study (see Fig 4). In meta-analysis, the pooled relative risk (RR) for low IQ in survivors of bacterial meningitis (any organism, excluding D’Angio et al. 1995) compared with controls was 4.99 (95% CI: 3.17, 7.86). Meta-analysis by organism was only possible for Hib studies: pooled relative risk for low IQ (excluding D’Angio et al. 1995) was not significantly different to zero: RR = 3.67 (95% CI: 0.63, 21.17).

#### Verbal IQ and performance IQ

**Fig 5:**
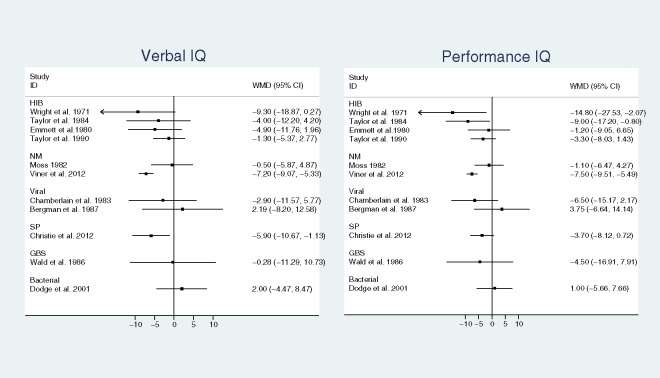
Forest plot of mean difference in Verbal and Performance IQ between meningitis survivors and controls by organism. Panel A shows Verbal IQ and Panel B shows Performance IQ. Abbreviations: *Haemophilus influenzae* b (Hib), *Streptococcus pneumoniae* (SP) *Neisseria meningitides* (NM), group B streptococcus (GBS); weighted mean difference (WMD)

Data on VIQ and PIQ were reported by 11 studies: 1 bacterial all-cause, 2 NM, 4 Hib, 1 SP, 1 GBS and 2 viral. Note that one study, Dodge et al. 2001, reported findings from younger and older children using different IQ tests which cannot be combined in meta-analysis: here we only include the larger sample of younger children. A further 4 included studies either provided insufficient data on VIQ or PIQ to review or used non-standardised measures. Fig 5 shows a Forest plot of mean difference in VIQ and PIQ between meningitis survivors and controls by organism. Across all bacterial causes, pooled mean difference between survivors and controls for VIQ was -3.73 (95%CI: -6.38, -1.09; I^2^ = 54%, p = 0.03) and for PIQ -4.38 (95% CI: -7.03, -1.73; I^2^ = 48%, p = 0.05). Meta-analysis by organism was only possible for Hib: pooled weighted mean difference for IQ was -3.20 (95% CI: -6.25, -0.15)) and PIQ (-5.41 (-10.12, -0.70).

#### Developmental delay

Twelve included studies reported developmental performance subsequent to meningitis; 3 studies reported on bacterial all-cause meningitis, 2 of neonatal meningitis, 4 for Hib, 1 for NM, 3 for SP, 1 for GBS and 3 viral (note some studies provided data for more than 1 organism). Six were case-control studies with the remainder being retrospective cohort studies. DD was reported using varying definitions of delay and using multiple different instruments. Seven DD studies were graded as providing level 3b evidence and 5 as level 4.

Table 2 summarises findings from the 12 studies by organism, showing differences in effect sizes between survivors and controls reported from case-control studies (as Cohen’s d) and the prevalence of DD in survivors from cohort studies. DD of approximately 0.5SD compared with controls was reported in the majority of case-control studies of bacterial causes, with the exception being the single Hib study (Wright et al. 1971) which reported no delay in cognitive development. Note that the sole neonatal bacterial study (Doctor et al. 2001) included only newborns with low birth weight (<1.5kg). Developmental domains assessed in bacterial studies varied across studies and it was not possible to summarize data by domain. No delay compared with controls was reported in either viral case-control study. Reported prevalence of DD in the retrospective cohort studies varied greatly from 0 to 35% across studies of bacterial causes. The only study of viral meningitis reported no identified DD across motor, language or cognitive development.

**Table 2:** Developmental delay (DD) by organism and developmental domain in CC and cohort studies.

	N of studies	CC studies: effect size (Cohen’s d) for DD compared with controls (Instrument used)	Cohort studies: prevalence of DD in meningitis survivors (Instrument used)
Bacterial all-cause	3	Dodge 2001: d = 0.5 delay (VSMS)	Singhi 2007: 34.5% (n = 20) with global developmental delay (DDST)
			Jiang 1990: DD in 17.4% (n = 8) (DDST)
Neonatal bacterial	2	Doctor 2001: cognitive delay d = 0.6, motor delay d = 0.5 (BSID)	Klinger 2000: 10% (n = 10) moderate or severe DD, defined as scores≥2 SD below mean (BSID)
SP	3		1. Goetghebuer 2000: 36% (n = 10) with cognitive delay and 76% (n = 16) with gross motor delay (DDST) 2. Jadavji 1986; 13.3% (n = 4) with DD defined as performance ≥2 months below chronological age (BSID) 3. Letson 1992: 10% (n = 1) with motor delay and 20% (n = 2) with language delay (various standardised tests)
HIB	4	Wright 1971: no differences in cognitive development (Bender-Gestalt & Frostig Developmental tests)	1. Goetghebuer 2000: 15% (n = 6) with cognitive delay; 19% (n = 5) with gross motor delay (DDST). 2. Letson et al. (1992): 14% (n = 6) with motor delay; 33% (n = 14) with language delay (various standardized tests) 3. Jadavji 1986: DD in 4% (n = 5) defined as ≥2 months below chronological age (BSID & VG).
NM	1		Jadavji 1986: Nil with DD defined as ≥2 months below chronological age (BSID & VG).
GBS	1	Wald 1986: visuo-motor integration delay d = 0.58 (various standardized tests)	
Viral	3	Bergman 1987: No differences in motor, language or cognitive development (various standardized tests)	Chang 2007: Nil with DD (DDST).
		Baker 1996: No delay in first 2 years post meningitis (BSID & PPVT)	
TB	0		

Table shows findings for DD assessed using validated standardised instruments from CC studies and from other studies. Findings for case control studies are shown as effect sizes expressed as Cohen’s delta (d) for the difference between cases and controls. Findings from other studies report proportions with DD. Only significant (p<0.05) differences are shown.

Abbreviations

CA: Chronological age

DDST: Denver Developmental Screening Test

BSID: Bayley Scale of Infant Development

PPVT: Peabody Picture Vocabulary Test

VSMS: Vineland Social Maturity Scale

VG: Vineland-Griffiths test

LBW: low birth weight, <1.5kg

d: Cohen’s D

Abbreviations: *Haemophilus influenzae* b (Hib), *streptococcus pneumoniae* (SP) *neisseria meningitides* (NM), *group B streptococcus* (GBS)

## Discussion

We found moderate evidence that surviving bacterial meningitis has a deleterious impact on IQ and development but no evidence that viral meningitis had an impact on IQ. Survivors of bacterial meningitis had an approximately 5 point reduction in IQ compared with healthy controls, a deficit that was similar across all organisms and equivalent to a 0.33 SD reduction in IQ. Survivors of bacterial meningitis were 5-fold more likely to have intellectual impairment (IQ<70) than controls. Studies of DD in younger survivors suggested an 0.5SD deficit that was largely consistent across bacterial causes, and was reported across multiple domains including cognitive, social, language and motor development.

Our findings suggest that bacterial but not viral meningitis results in a leftward shift of the IQ distribution, lowering mean IQ and increasing the proportion with moderately severe intellectual impairment and DD. The impact of meningitis appeared to be of a similar order on VIQ and PIQ, suggesting a broad non focal impact upon cognitive function similar to the effects of non-specific brain injury. This is also supported by the broad range of domains reported to have developmental delays in differing studies. Whilst it is notable that mean IQ in survivors remained in the normal range across all organisms, an 0.33SD reduction is likely to be highly meaningful for those with premorbid IQ in the lower part of the IQ distribution. Those with low IQ or moderately severe intellectual impairment are likely to have much poorer educational attainments, poorer later employment opportunities and a range of poorer health outcomes.

We found no high quality studies of the cognitive outcomes of TB, fungal or parasitic meningitis. We found little evidence that neonatal meningitis had a greater impact than meningitis later in early childhood, with estimates for the relative risk of low IQ similar to those for meningitis in later childhood. Given that only two studies specifically examined periods after early childhood, we could not examine whether meningitis in childhood had a greater or lesser impact on outcomes. Data limitations meant that we were not able to examine the impact severity of meningitis on outcomes.

### Comparison with literature

No previous studies have systematically reviewed the impact of meningitis on mean full-scale IQ or its verbal and performance components. Our finding that the pooled mean prevalence of low IQ i.e. IQ <70 from bacterial causes was approximately 5% is lower than a previous estimate of 9.1% by Edmond et al. 2010,[4] however that study provided no details on how the quality of IQ data in included studies was assessed and likely included studies considered ineligible by our review. Previous systematic reviews have suggested that the prevalence of major sequelae is highest after SP meningitis,[4] however we were unable to confirm this largely due to lack of high quality SP studies. No previous systematic reviews have examined the impact of viral meningitis on IQ.

### Strengths and limitations

We undertook an extensive systematic review using search terms similar to a previously published systematic review[4] and undertook thorough hand-searching of reviewed articles. We included only studies which used validated measures of intelligence or early childhood development and which reported adequate data to allow assessment. Random effects meta-analyses were undertaken to allow for significant heterogeneity seen in many meta-analyses.

These data are subject to a number of limitations. The great majority of studies were underpowered. A number of studies, including those some of potentially high quality, were excluded as they only reported findings for sub-groups of survivors. There were few population-based studies, with the great majority being hospital series and thus open to recruitment bias. In some included studies, the sample that underwent cognitive testing was a subset of the overall group of survivors, and this is likely to be a further source of bias. The direction of such biases is unclear, as survivors with major cognitive deficits were potentially less likely to be recruited, or conversely, parents of children more severely affected by meningitis may have been more active in seeking recruitment to studies. Data on DD were particularly diverse, with studies using different instruments and reporting across different domains, making findings difficult to synthesize. Data were insufficient to summarize by developmental domain. Because of this we believe that our systematic review only provides moderate evidence for the effects of meningitis on IQ and development.

### Conclusions

We found moderate evidence that bacterial meningitis is associated with significant and clinically meaningful reductions in overall, verbal and performance IQ and with meaningful developmental delay in survivors compared with healthy controls. We found no effects of viral meningitis on IQ or developmental progress. We found data quality of studies to be limited. Further work is needed to robustly evaluate the sequelae of differing forms of meningitis, particularly in low-income countries. Survivors of bacterial should be routinely offered screening for cognitive deficits and developmental delay in addition to hearing loss. These data will inform cost-effectiveness assessments of future vaccines and planning of service provision for survivors.

## Supporting information

S1 Checklist PRISMA 2009 checklist. (DOC)

S1 Table Characteristics of reviewed studies. (DOCX)
