# Supplementary material for: Impact of meningitis on intelligence and development: A systematic review and meta-analysis
Source: PLoS One. 2017 Aug 24;12(8):e0175024. doi: 10.1371/journal.pone.0175024 (PMC5570486; doi:10.1371/journal.pone.0175024)
Supplement: S1 Table — (DOCX) [file pone.0175024.s002.docx]

# S1 Table. Characteristics of reviewed studies

| **Author** | **Reference** | **Study type** | **Country** | **Organism** | **Sample (case / control)** | **Age at meningitis**  **Months(m) or Years(y)** | **Age at assessment**  **Months(m) or Years(y)** | **Case Recruitment** | **Control: recruitment (R) & matching (M)** | **IQ Instrument** | **FSIQ mean** | **low IQ (<70) %** | **VIQ** | **PIQ** | **Developmental delay** | **Quality** |
| --- | --- | --- | --- | --- | --- | --- | --- | --- | --- | --- | --- | --- | --- | --- | --- | --- |
| Anderson et al. | Journal of Pediatric Psychology 29(2) pp. 67–81, 2004 | CC | Australia, Melbourne | Bacterial | 107/96 | 3-79m | 10-18y | Hospital series | R: school  M: school grade, sex | WISC & WAIS | 97.20 |  |  |  |  | 3b |
| Baker et al. | Clin Pediatr (Phila). 1996 Jun;35(6):295-301 | CC | USA, Ohio | Viral | 8/13 | <3m | 3y | Hospital series | R: Private clinics  M: age, sex, SES, race | Stanford Binet | 104.00 |  | Yes | Yes | BSID & PPVT. No delay in first 2 years post meningitis. | 3b |
| Bergman et al. | J Pediatr. 1987;110(5):705-9 | CC | USA, Pittsburg | Viral | 33/31 | 0-12m | 3-17y | Hospital series | R: sibling  M: near age, sex | WISC, WPPSI & WAIS | 102.80 | 3.0% | Yes | Yes | range of standardised tests: No differences in motor, language or cognitive development | 3b |
| Chamberlain et al. | Child Care Health Dev. 1983 Jan-Feb;9(1):29-47 | CC | UK (England and Wales) | Viral | 49/45 | 0-12m | 5.7-8.8y | Population -based | R: school  M: age, sex | WISC | 97.90 |  | Yes | Yes |  | 3b |
| Christie et al. | Pediatrics. 2009 Mar;123(3):e502-9 | CC | UK, Oxford and North Thames | SP | 97/93 | 0-15m | 3-20y | Population-based | R: sibling or neighbour  M: age, sex | WPPSI, WASI | 102.40 |  | Yes | Yes |  | 3b |
| D'Angio et al. | Arch Pediatr Adolesc Med. 1995 Sep;149(9):1001-8. | CC | USA: Navajo Indians | HIB | 41/79 | 3-23m | 49-102m | Population-based | R: 2 groups: 38 sibling and 41 hospital  M: age, sex for both groups | Stanford-Binet | 79.00 | 24.4% |  |  |  | 4 |
| Doctor et al. | Clin Pediatr (Phila) 2001; 40; 473 | CC | USA, Ohio | Neonatal Bacterial all cause | 39/2053 | 0-1m | 20m | Hospital series | R: hospital  M: all other very low-birthweight infants admitted during same period | BSID |  | 38.5% |  |  | BSID in LBW: cognitive delay d=0.6, motor delay d=0.5 | 3b |
| Dodge et al. | J Child Neurol. 2001 Nov;16(11):854-7. | CC | USA, Missouri | Bacterial | 185/116 | 2-168m | 5.3-7.5y | Hospital series | R: sibling  M: nearest age, sex | WPPSI, WISC | 99.00 |  | Yes | Yes | Vineland Social Maturiy Scale was significantly lower in cases than in controls (113 vs 124, p<0.05). VSMS: D=0.5SD delay | 3b |
| Emmett et al. | Aust Paediatr J. 1980 Jun;16(2):90-3 | CC | Australia, Brisbane | HIB | 14/14 | 9-72m | 8y | Hospital series | R: siblings  M: nearest age, sex | WISC | 103.20 |  |  |  |  | 3b |
| Fellick et al. | Arch Dis Child 2001;85:6-11 | CC | UK, Liverpool | NM | 115/115 | 1-180m | 8.8-25y | Hospital series | R: primary care doctor  M: age, sex | WISC | 92.90 |  | Yes | Yes |  | 3b |
| Franco SM | Am J Dis Child. 1992 May;146(5):567-71 | CC | USA, Kentucky | Neonatal Bacterial all cause | 15/14 | 0-1m | Child | Hospital series | R: neonatal unit  M: age, sex, birthweight, race | Stanford Binet |  | 26.7% |  |  |  | 3b |
| Jiang et al. | Developmental medicine and child neurology 1990; 32(6): 473-480 | CC | China, Shanghai | Bacterial | 46/76 | 0-48m | 2-6y | Hospital series | R: hospital clinic  M: similar age | Peabody Picture Vocabulary Test | 96.00 |  |  |  | Denver Developmental Screening Test (DDST) or PPVT: DD in 17.4% (n=8) | 3b |
| Merkelbach et al. | Acta Neurol Scand 2000: 102: 118-123 | CC | Germany | Bacterial | 22 /17 | Adults | 36-69y | Hospital series | R: volunteers  M: age, sex | German WAIS | 110.00 |  |  | Yes |  | 3b |
| Moss PD | Arch Dis Child 1982;57:616-21 | CC | UK, Bolton | NM | 60/60 | 1-96m | Child | Population-based | R: school  M: grade, sex | WISC | 94.00 |  |  | Yes |  | 4 |
| Saha et al. | Clin Infect Dis. 2009 Mar 1;48 Suppl 2:S90-6 | CC | Bangladesh | SP | 51/51 | 2-59m | 4-18m | Hospital series | R: population  M: age, sex, SES | Stanford Binet |  | 41.2% |  |  |  | 3b |
| Salih et al. | Scand J Infect Dis. 1991;23(2):175-82 | CC | Sudan | Bacterial | 19/19 | 0-82m | 24-84m | Hospital series | R: sibling  M: nearest age | Stanford Binet | 92.30 | 10.5% |  |  |  | 4 |
| Sell et al. | Pediatrics 1972;49:212-7 | CC | USA, Vanderbilt | HIB | 21/21 | 2-36m | Mean 8 years | Hospital series | R: siblings  M: nearest age | WISC | 86.00 | 19.0% |  |  |  | 3b |
| Stevens JP et al. | Arch Dis Child Fetal Neonatal Ed 2003;88:F179-F184 | CC | UK (England and Wales) | Bacterial | 111/162 (113 hospital; 49 population-based) | <1m | Mean 9.4y | Population-based | R: 2 groups: hospital or population-based  M: age, sex for both groups | WISC | 88.8 | 13.5% | Yes | Yes |  | 3b |
| Taylor et al. | New Engl J Med 1990;323:1657-63 | CC | Canada | HIB | 97/97 | 0-156m | 9.6y | Hospital series | R: sibling  M: nearest age | WISC | 108.00 | 1.0% |  | Yes |  | 3b |
| Taylor et al. | Pediatrics 1984:74(2):198-205 | CC | USA, Pittsburg | HIB | 24/24 | 3-36m | 7-11y | Hospital series | R: sibling  M: nearest age | WISC | 96.70 |  |  | Yes |  | 3b |
| Tejani et al. | Dev Med Child Neurol 1982 Jun;24(3):338-43. | CC | USA, New York | HIB | 8/8 | 2-24m | Child | Hospital series | R: sibling  M: nearest age | WPPSI | 97.00 |  |  |  |  | 3b |
| Viner et al | Lancet Neurology 2012; 11(9):774-83 | CC | England | NM | 246/328 | 1-156m | 3-16y | Population-based | R: population-based  M: age, sex, SES | WPPSI, WISC | 99.8 | 0.8% | Yes | Yes |  | 2b |
| Wald et al. | Pediatrics. 1986 Feb;77(2):217-21 | CC | USA, Pittsburg | GBS | 18/21 | 0-6m | 8.6y | Hospital series | R: sibling  M: nearest age | WISC | 101.90 |  |  | Yes | various standardized tests: visuo-motor integration delay d=0.58 | 3b |
| Wright et al. | J Abnorm Psychol. 1971 Apr;77(2):181-3. | CC | USA, Oklahoma | HIB | 10/10 | 3-70m | 6-15y | Hospital series | R: not stated  M: age, sex, SES | WISC | 92.60 |  |  | Yes | Bender-Gestalt & Frostig Developmental tests: no differences in cognitive development | 3b |
| Borg et al. | Pediatrics 2009 Mar;123(3):e502-9. | CC | UK (England and Wales) | NM | 101/101 | 13-19 years | 16-22y | Population-based | R: population-based  M: age, sex, SES | WAIS | 102.10 |  |  |  |  | 2b |
| Chin et al. | J Pediatr. 1985 May;106(5):819-22 | RC | Canada, Toronto | GBS | 20 | <1m | 1.5-7y | Hospital series |  | WPPSI, WISC |  | 15.0% |  |  |  | 4 |
| Fitzhardinge et al. | Dev Med Child Neurol. 1974 Feb;16(1):3-8 | RC | Canada, Montreal | Neonatal Bacterial all cause | 18 | <1m | 1-11y | Hospital series |  | Stanford Binet, WPPSI, WISC | 90.00 | 16.7% |  |  |  | 4 |
| Gade et al. | Developmental Neuropsychology 1992;8(4):447-57 | RC | Denmark, Copenhagen | SP | 33 | 6-15 years | Mean 12.1y | Hospital series |  | WISC | Not reported |  | Yes | Yes |  | 4 |
| George et al. | Indian Pediatrics 2002; 39:663-667 | RC | India | Bacterial | 100 | 0-60m | 4-11y | Hospital series |  | Binet Kammath Test-Indian version of Stanford-Binet |  | 6.0% |  |  |  | 4 |
| Goetghebuer et al. | Tropical Medicine and International Health 2000; 5(3): 207–213 | RC | The Gambia | HIB & SP | HIB: 42 SP: 31 | 0-144m | Child | Hospital series |  |  |  |  |  |  | DDST SP: 36% (n=10) cognitive delay and 76% (n=16) gross motor delay HIB: 15% (n=6) cognitive delay; 19% (n=5) gross motor delay | 4 |
| Hutchison et al. | Can Med Assoc J. 1963 Jul 27;89:158-66. | RC | Canada, Winnipeg | Bacterial | 41 | 6m to adult | NR: child and adult | Hospital series |  | Stanford Binet |  | 7.3% |  |  |  | 4 |
| Jadavji et al. | Pediatrics 1986;78:21-5 | RC | Canada | Bacterial | SP: 30 HIB: 124 NM: 17 | 0m-18 years | Mean 26m | Hospital series |  |  |  |  |  |  | BSID & VG SP: 13.3% (n=4) DD defined as performance ≥2 months below CA HIB: BSID & VG: DD 4% (n=5) ≥2 months below CA. NM: Nil with DD | 4 |
| Klinger et al | Pediatrics 2000;106;477-482 | RC | Canada | Neonatal Bacterial all cause | 101 | <1m | Mean 12m | Hospital series |  |  |  |  |  |  | BSID: 10% (n=10) moderate or severe DD, defined as scores≥2 SD below mean | 4 |
| Letson et al. | Am J Dis Child. 1992 May;146(5):560-6 | RC | USA, Alaska | HIB & SP | HIB: 43 SP: 10 | 1-30m | child | Population-based |  | WISC |  | HIB: 11.6% SP: 10.0% |  |  | HIB: various tests: Alaskan native infants; 14% (n=6) motor delay; 33% (n=14) language delay. SP: various standardised tests: Alaska Native infants 10% (n=1) motor delay and 20% (n=2) language delay | 4 |
| Lindberg et al. | Pediatrics. 1977 Jul;60(1):1-6 | RC | Sweden | HIB | 82 | 2m-22 years | NR: child and adult | Hospital series |  | WISC |  | 1.2% |  |  |  | 4 |
| Schoeman et al. | Developmental Medicine & Child Neurology 2002, 44: 522–526 | RC | South Africa, Cape Town | TB | 76 | 3m-13 years | 7-19y | Hospital series |  | WISC |  | 43.4% |  |  |  | 4 |
| Singhi et al. | Indian J Pediatr 2007;74:369-374 | RC | India | Bacterial | 80 | 2-144m | 14-189m | Hospital series |  | Denver Development Screening Test (DDST) |  |  |  |  | DDST: 34.5% (n=20) with global developmental delay | 4 |
| Todd et al. | Arch Dis Child. 1964 Jun;39:213-25. | RC | UK, Liverpool | TB | 65 | 0-15 years | 6-27y | Hospital series |  | Stanford Binet, WISC |  | 9.2% |  |  |  | 4 |

Table abbreviations:

Study Type: CC: case-control; RC: retrospective cohort

Age at assessment: This is noted as child when specific ages in childhood not reported; Mean reported where age range not stated; NR: not reported.

Controls: Recruitment (R), Matching (M), socioeconomic status (SES).

IQ: FSIQ: full-scale IQ; VIQ: verbal IQ; PIQ: performance IQ

Y: yes, N: no

IQ assessment methods: WISC: Weschsler Intelligence Scale for Children; WIPPSI: Wechsler Preschool & Primary Scale of Intelligence; WAIS: Wechsler Adult Intelligence Scale; Stanford Binet: Stanford Binet Intelligence Scale; BSID: Bayley Scales of Infant Development

Developmental delay assessment methods: BSID: Bayley Scales of Infant Development; KABC: Kaufman Assessment Battery for Children;

DDST: Denver Development Screening Test;

*Haemophilus influenzae b* (Hib), *Streptococcus pneumoniae* (SP) *Neisseria meningitides* (NM), group B streptococcus (GBS).
